# Aphonia of Twenty Months Standing, Relieved by Iodine Inhalation

**Published:** 1853-09

**Authors:** Edward B. Stevens

**Affiliations:** Lebanon, O.


From the Western Lancet.

*Aphonia of Twenty Months Standing, Relieved by Iodine Inhalation.* By EDWARD B. STEVENS, M.D., Lebanon, O.

IN a communication to the American Medical Association, in its Vol. of Transactions for 1850, Prof. Pancoast has given the record of two cases of loss of voice. The one of six, the other of seven months standing, both cured by inhalation of a dilut. chlorine vapor.

In connection with these cases, Dr. Pancoast remarks: "The form of aphonia here alluded to, is that which many practitioners must have met with, following an ordinary cold, without leaving any perceptible organic lesion in the pulmonary apparatus. The The voice is reduced to a faint hoarse whisper, distinguishable only at a distance of a few feet, and on continued attempt to talk, though it gives no pain, becomes quickly attended with a feeling of fatigue, as though there was some obstruction to the passage of air through the larynx. In breathing merely, there is little or no difficulty, in these cases, as the individuals are capable of undergoing considerable exertions without very unusual signs of fatigue. The difficulty has appeared to me to be in the paralyzed condition of the muscles of the larynx, whose business it is to dilate the rima glottidis, during the act of articulation."

The conclusion of Dr. Pancoast is, that such agent as will excite a healthy and proper degree of stimulation in the affected structure ought rationally to restore the power of articulation. He consequently used the dilut. chlorine vapor, with entire success in the two cases referred to,—at the same time suggesting that iodine, or other similar agents would doubtless produce a similar effect.

The following case of this kind lately occurred in my practice, chiefly remarkable from the long duration of absence of voice, being twenty months, in other respects similar to those related by Dr. Pancoast.

*April* 6, 1853.—Miss——— applied for medical advice and treatment, in a case of loss of voice, of twenty months standing, supervening upon a slight attack of influenza. Has been subject to brief attacks of hoarseness, lasting for a few days at a time, for several years. General health delicate. Since the present attack, has been subject to a great variety of treatment, including the application of nit. silv. in strong solution, within the larynx by means of the sponge probang. Nothing, however, producing any effect upon the voice. I find upon careful examination, no especial evidence of disease in the fauces: there is an entire inability to produce sound of any description with the proper vocal organs; all attempts at speaking are made with the lips—*whispering*. But not being able to divest myself of the idea that a follicular inflammation of the throat and bronchial tubes was the cause of the mischief in some way, I commenced the treatment by directing the inhalation of nit. silv. prepared with the lycopodium, as an inpalpable powder, and inhaled by means of the apparatus introduced by Dr. Ira Warren. This treatment was faithfully persevered in for one month, with no better results than the previously tried remedies.

*May* 7.—Acting upon the idea suggested by Prof. Pancoast of paralysis of the muscles of the larynx, I now determined to try the iodine vapor. I accordingly selected an apparatus, consisting of a metalic vase or urn, with a close fitting cover, flexible tube, and mouth piece attached, (used some years since for breathing medicated vapors in the treatment of consumption), and directed my patient, after filling the vessel half full with hot water, to drop in 20 drops *tinct. iodine*, and inhale the vapor produced by the heated water. Inhalation to be repeated once or thrice daily, according to the irritation or effects, otherwise, produced. The first inhalation produced great nausea for a short time, and copious bloody expectoration, but accompanied by an almost immediate, though partial restoration of voice. The dose of iodine was directed to be reduced to 15 drops; and thereafter no unpleasant effects were produced. The voice continued to improve steadily nnder this treatment, until the end of a week it had acqured the natural fullness and distinctness of tone.

*June* 15.—More than a month has elapsed since the restoration of voice ; it continues distinct and natural.

